# General practitioner and nurse experiences of type 2 diabetes management and prescribing in primary care: a qualitative review following the introduction of funded SGLT2i/GLP1RA medications in Aotearoa New Zealand

**DOI:** 10.1017/S1463423624000264

**Published:** 2024-09-16

**Authors:** Kimberley Norman, Sara Tareq Mustafa, Shemana Cassim, Hilde Mullins, Penny Clark, Rawiri Keenan, Leanne Te Karu, Rinki Murphy, Ryan Paul, Tim Kenealy, Lynne Chepulis

**Affiliations:** 1 School of Primary and Allied Health Care, Monash University, Melbourne, Australia; 2 Medical Research Centre, Te Huataki Waiora School of Health, University of Waikato, Hamilton, New Zealand; 3 School of Psychology, Massey University, Auckland, New Zealand; 4 Department of Nursing, Te Huataki Waiora School of Health, University of Waikato, Hamilton, New Zealand; 5 Northcare Medical Centre, Hamilton, New Zealand; 6 Faculty of Medical and Health Sciences, University of Auckland, Auckland, New Zealand; 7 Te Whatu Ora Health New Zealand, Hamilton, New Zealand

**Keywords:** medication, primary health care, qualitative research, type 2 diabetes

## Abstract

**Aim:**

To explore the views of general practitioners (GPs) and nurses on type 2 diabetes (T2D) management, including the use of recently funded T2D medications in New Zealand (NZ) and their perceived barriers to providing optimal care.

**Background:**

T2D is a significant health concern in NZ, particularly among Māori and Pacific adults. Characterised by prolonged hyperglycaemia, T2D is generally a progressive condition requiring long-term care.

**Methods:**

Semi-structured interviews were conducted between July and December 2022 with 21 primary care clinicians (10 GPs and 11 nurses/nurse prescribers) from nine different general practice clinics across the Auckland and Waikato regions of NZ. Framework analysis was conducted to identify common themes in clinicians’ perceptions and experiences with T2D management.

**Findings:**

Three themes were identified: health-system factors, new medications, and solution-based approaches. Lack of clinician time, healthcare funding, staff shortages, and burn-out were identified as barriers to T2D management under health-system factors. The two newly funded medications, empagliflozin and dulaglutide, were deemed to be a positive change for T2D care in that they improved patient satisfaction and clinical outcomes, but several clinicians were hesitant to prescribe these medications. Participants suggested that additional education and specialist diabetes support would be helpful to inform optimal medication prescribing and that better use of a multi-disciplinary team (clinical and support staff) could support T2D care by reducing workload, addressing cultural gaps in healthcare delivery, and reducing burnout. An improved primary care work environment, including appropriate professional development to support prescribing of new medications and the value of collaboration with a non-regulated workforce, may be required to facilitate optimal T2D management in primary care. Future research should focus on interventions to increase support for both clinical teams and patients while adopting a culturally appropriate approach to increase patient satisfaction and improve health outcomes.

## Introduction

Type 2 diabetes (T2D) is a significant health issue globally, affecting nearly half a billion people worldwide (Khan *et al.*, 2020). Of concern, prevalence continues to rise, and people are increasingly being diagnosed at a younger age. In Aotearoa New Zealand (NZ), T2D affects more than 300,000 adults, including a disproportionate number of Māori (the Indigenous people of NZ) and Pacific peoples (Te Whatu Ora Health New Zealand, 2023).

Māori experience inequity in healthcare as evidenced by higher rates of comorbidities (age-standardised relative risk of 2.7–3.2 for congestive heart failure, obesity, and complicated diabetes) and a shorter life expectancy (73/77 years vs 81/84 years for males/females) compared to non-Māori in NZ (Gurney *et al*., 2020). For many, this health inequity results from social deprivation and the ongoing effects of colonialism (e.g. a lack of healthcare delivery that encompasses a Maori worldview/cultural practices; continued racism and social deprivation; Moewaka Barnes & McCreanor, 2019; Reid *et al.*, 2019) such that Māori often have reduced engagement with healthcare services and access to medications and medical information (Palmer *et al*., 2019). Further, Māori patients have reported a lack of strong clinician-patient relationships as one of the main factors hindering their healthcare (Palmer *et al*., 2019).

Although complex, T2D can be effectively managed through lifestyle and behavioural change in combination with glucose-lowering therapies. Optimal management needs to be collaborative between both the patient and the healthcare team, though many patients with T2D continue to have suboptimal glycaemic control (Chepulis *et al*., 2021). Historically, use of diabetes medications in NZ has been suboptimal, centred around the use of metformin, sulphonylureas, and insulin (Krebs *et al*., 2016). However, more recently in NZ, additional agents have become available in 2021, including sodium-glucose cotransporter-2 inhibitors (SGLT2i) and glucagon-like peptide 1 receptor agonists (GLP1RAs) funded for use in patients with T2D in NZ. This is significant as these two classes of medications have been available overseas for more than 10 years and offer significant advantages over other medications for T2D, including weight loss, reduced progression of cardiovascular-renal diseases, and they do not cause hypoglycaemia alone (Vaduganathan *et al*., 2018). Funded access to these agents is also prioritised for Māori and Pacific communities (who can access these agents if they have established T2D with an HbA1c above 53 mmol/mol [7%]; whilst those of other ethnicities must also have clinical indication of cardiovascular and/or renal disease), with the aim of reducing health inequity (Pharmac Te Pataka Whaioranga, 2021). Early research suggests that there have been higher rates of therapy initiation in these groups in NZ compared to other countries, with nearly half of all eligible Māori patients with T2D prescribed these agents during 2021–2022 (Paul *et al*., 2023) compared to less than a quarter of patients in international studies (Arnold *et al*., 2022).

In NZ, primary care is funded by a partial fee-for-service payment and patient co-payments. Primary care encompasses a range of general practitioners (GPs), nurses, and nurse prescribers/advanced practitioners, as well as additional support from pharmacists and nutritionists as required. Much of the remit of diabetes care comes under the scope of nurse practice; however, there is concern that clinicians are not meeting the needs of many patients in NZ at present, due to the current workforce issues and a deficit of at least 8,000 clinical staff (Whitebird *et al*., 2017; Nicholls *et al*., 2021; Bell *et al*., 2021).

Further, it is acknowledged that primary care clinicians experience barriers to providing optimal diabetes healthcare (Simmons *et al*., 1998; Chepulis *et al*., 2021). NZ clinicians have previously reported that limited clinician availability, access and funding restraints, lack of T2D specialists in primary care, and inadequate training in cultural safety can all impact the delivery of T2D care (Simmons *et al*., 1998; Chepulis *et al*., 2021); and this is similar to that seen internationally (Jones *et al*., 2014; Rushforth *et al*., 2016). However, we know little about how the current workforce shortages impact the current clinical experiences and prescribing behaviours, particularly following the recent funding of SGLT2i and GLP1RA medications for people with T2D in NZ. Thus, the aim of this study was to explore the views of GPs and primary care nurses about their experiences of effective T2D management in their practices, including the use of SGLT2i/GLP1RA medications and their perceived barriers to providing optimal T2D care.

## Materials and methods

### Study setting and participant recruitment

This study was part of a larger project to explore the role of health-system factors on T2D care, with this study focussing on clinicians’ experiences. Clinicians were recruited between July and December 2022 from a total of nine general practices and Māori health providers in the Auckland and Waikato regions of NZ (Māori health providers offer primary care services but also include Māori practice ownership and/or governance). Participants were eligible if they were practising GPs or nurses and actively involved in delivering care to patients with T2D. The research team circulated an email invitation to their networks within primary care. This email detailed the research objectives and invited participants to contact the researcher to discuss further or volunteer to participate. A snowballing strategy (Emmel, 2013) was utilised whereby early participants and primary care contacts also forwarded the email to their own networks in primary care within the Auckland and Waikato regions, with the aim of recruiting participants who engage with T2D patients on a regular basis. Prior to data collection, all eligible participants were provided a copy of the participant information sheet and the consent form. All participant questions and concerns were answered prior to obtaining written consent. Participants were offered a $50 voucher as koha (gratitude) to thank them for their time.

### Data collection

Participant data were collected by KN via semi-structured interviews to allow participants to guide the narrative while enabling the interviewer to prompt and ask follow-up questions (Ahlin, 2019). Aligning with best methodological practice (Magnusson & Marecek, 2015), the semi-structured interview guide was developed by the research team, which comprised expert early-, mid-, and late-career research academics, general practice clinical experts, and a Māori Advisory Group, all working, practicing, or researching in this space. These included open-ended questions including ‘please tell me about your experience with delivering type 2 diabetes healthcare in your practice?’, ‘could you please tell me your experience with using the recently approved diabetes medications SGLT2i and GLP1RA?’, and ‘please tell me about any difficulties you have with delivering effective type 2 diabetes healthcare in your role?’. Questions aligned with the significant topic areas and gaps identified from a thorough literature review and reflection on the larger research project. While specifically including questions on clinician experience, the new medications and barriers to delivering T2D healthcare, the participants were encouraged to speak about other factors important to their experience. All interviews were conducted via Zoom due to the ongoing effects of the COVID-19 pandemic. At the beginning of each interview, the objective of the study and participants’ rights were re-stated. Before the interview, all participants were offered the opportunity to open the engagement in a culturally appropriate manner, such as reciting a prayer or Karakia (Māori spiritual time of connection). Participants were encouraged to share as much about their experience for as long as they wanted to. Interviews were audio-recorded and lasted between 20 and 70 minutes.

### Analysis

All interview recordings were transcribed verbatim using a transcription software (otter.ai) and checked for accuracy by a researcher. Each transcript was read and re-read by three researchers (KN, SC, and HM) to facilitate immersion in the data. The transcripts were analysed using a framework matrix previously used in a multi-disciplinary healthcare setting (Gale *et al*., 2013). A study-specific framework relating to the key aims of the study, alongside the existing literature and its gaps, was then created through an iterative process by the three researchers who led the analysis (and based on the framework of Gale *et al*., 2013). The framework comprised points around clinician experience of delivering T2D healthcare, clinician views on the newly funded T2D medications, the perceived barriers to delivering T2D healthcare, and ways that T2D intervention or management can be improved for future healthcare. Each transcript was printed out and coded in the left-hand margin using this framework. All sections of discourse were included if related to any of the above categories. In the right-hand margin, researchers coded for anything that was significant to the participants’ narrative relating to T2D healthcare delivery. This enabled new, additional, or unexpected concepts to be identified and highlighted that were significant to the clinician’s perspective that did not fit into the framework. To ensure a rigorous analysis process, each transcript was analysed in turn, and then comparatively re-analysed. Preliminary analysis found significant data focused on barriers to effective T2D delivery for clinicians (from individual, practice and health-system levels), varying views on the new medications, significant cultural barriers, and the complexities of diabetes treatment requiring effective lifestyle changes along with medication adherence. These preliminary concepts were re-analysed by three researchers, followed by debate and discussion with the wider multi-disciplinary team, resulting in three main themes being finalised. While data saturation is considered to be situated and subjective (Braun & Clarke, 2021), the researchers found no new consistent themes emerging across narratives.

### Ethical approval

This study was approved by the University of Waikato Human Research Ethics Committee (HREC(Health)2022#19).

## Results

### Participant characteristics

A total of 21 clinicians from nine clinics participated in the study. Of the 21 clinicians, ten were GPs (three female and seven male) and 11 were nurses/nurse prescribers (10 female, one male). Participants were between the ages of 25 and 55 years and identified as Māori, NZ European, English (United Kingdom), South African, Indian, Korean, and Chinese.

### Themes

Three overarching themes were identified, two from the framework analysis guide and one from outside the guide. These were: barriers to effective T2D management, clinician perspectives on the new medications, and the potential benefits of extending the concept of a multi-disciplinary team for T2D patients.

### Barriers to delivering effective T2D healthcare

Participants discussed various health-system-related factors that hindered their role and ability to adequately manage and/or prescribe for patients with T2D, including limited appointment times and lack of funding, which led to work dissatisfaction. Many participants indicated that the limited time clinicians had for patient consults was a significant barrier:

‘*But yeah, for me, it’s always been time. It’s just there’s not enough time to, um yeah, that’s… And I guess, loads of other barriers, but that’s the biggest one for me’.* (GP-04)

Another clinician identified that particularly for those with comorbidities, more time was needed than the standard 15-minute appointment:

*‘This is just…. what I want to do with my patient partly because chronic care patients, you know, most of the time, by the time they reach 60 [years], they have five or six conditions, and they [have] different health conditions. And we only got 15 minutes for them, they come with a problem, and we deal with the problem and ignore the rest of it, and we kick them out’.* (GP-01)

Health-system staff shortages were also highlighted as hindering the ability to provide quality diabetes care to patients:

‘*But just any staff at all would be good right now. We don’t even have appointments available. We’re only working- we’ve only got enough nurses to staff urgent care. So, you can’t even get an appointment and have a chat if you were diabetic … Like, its dire at the moment’.* (Nurse-07)

Wider concepts including a lack of funding for the primary care sector to operate were also cited as a factor for the strain on the health system. Participants indicated that inequitable distribution of funding in the healthcare system has led to an increased burden on primary care and their role as clinicians:

*‘I guess it will probably…. just comes down to funding, giving us more time. I feel like I’m doing not as good a job as I would like to, or providing care as I would like to, because there are external limitations that are put on’.* (GP-10)

*‘I think definitely, as always gonna be more funding needed, you know, less managers more workers on the floor… Because it does seem to get stretched a lot, you know, the whole health dollar. And a lot more stuff has been pushed out to us as practices’.* (Nurse-01)

Due to the limitations in the healthcare system, some clinicians had also expressed difficulties in delivering effective T2D healthcare including factors, such as work dissatisfaction and burn-out, which impacted the care they provided patients in their role:

*‘So, I feel like I’m um, you’re providing suboptimal care sometimes. And that’s, I think, also contributing to a lot of burn-out for my colleagues as well. So they come in to general practice to do this much but in reality we can only provide maybe 50% of what we can do. I think that’s a real difficulty that everyone faces’.* (GP-10)

*‘I don’t think there’s a quality consult, you know. It’s all we’ve got; it’s all I can do. I feel stink, really. Yeah. I know I’m not meeting their needs in that time frame’.* (Nurse-06)

Clinicians also noted difficulties in delivering T2D care over extended periods of time with some patients who were dealing with other health factors such as unstable housing. One clinician highlighted:

*‘But it [the system] is set up in such a way that it favours people who have an address. It’s another problem for our patients. We have a high number of people in emergency housing, they’re moving all the time, we struggled to keep track of applications because they’re always moving their housing situations. And so phone numbers often drop out, and you don’t get the updated phone number. So, getting hold of people is hard I’m going through my recalls for smoking and diabetes, cardiovascular risk systems at the moment. And say about one in four people that I tried to contact, the phone numbers it brings to a dead dial tone. Or it’s completely disconnected’.* (GP-02)

Clinicians have noted the lack of culturally appropriate T2D care, as well as language barriers, which hindered their ability to accommodate ethnic diversity across patients:

*‘Because I speak Mandarin, so a lot of my patients, these majority are, you know, Chinese and Mandarin speaking only. So, there is, I do definitely have a group of patients that they just, they don’t, there’s a big fear in terms of what diabetes means and having to take Western medication. So, it takes years to kind of gradually come get a bit of buy in’.* (GP-04)

### Clinician perspectives on newly introduced medications

There were varying views about the newly introduced T2D medications to NZ. Many clinicians reported positive experiences with the prescribing of SGLT2i and GLP1RA agents, and satisfaction with these drugs now being available for them to prescribe to their patients. Two clinicians expressed that the medications demonstrated clinical improvements and overall patient satisfaction:

*‘So, since they’ve been funded, it’s just huge, you know, actually seeing a proper reduction of HbA1c and weight loss in patients. And, you know, patients, by and large tolerate it really well. And so greater buy in, and I’m actually seeing improvement in kidney function. Whereas before we just rolled over, and it’s just that much of the same and not really getting anywhere’.* (GP-04)

*‘They [patients] quite like the idea that it’s a new medication that’s available and it’s got the kind of added advantages of other cardioprotective and things like that. And the ones that I’ve personally put on dipeptidyl peptidase 4 inhibitors [vildagliptin] or SGLT2i [empagliflozin], they’ve actually made really good progress on the HbA1c. And so, I think when the patients see the numbers, they are more motivated to continue with the medication as well’.* (GP-09)

However, some clinicians struggled with staying up to date with the current best practice in T2D management. Whilst many participants were aware of the diabetes education course that was available online during 2021–2022 (offered by Dr XXX, endocrinologist), many also indicated that additional support from a diabetes specialist or other reliable sources (e.g., Ministry of Health, NZ Society for the Study of Diabetes) is vital to support clinicians to confidently prescribe the new medications. One clinician highlighted that having a specific T2D nurse available was useful:

*‘We’re lucky that we have a diabetic nurse that kind of helps us, and she does all the educating and I just have to, you know most of the time, to prescribe and talk about the side effects. But especially um, so I guess the newer medications, so the, like the Jardiance [empagliflozin] and Trulicity [dulaglutide], gathering experience are not quite… I wouldn’t be… I’m not fully confident, but I am increasingly prescribing’.* (GP-04)

Two clinicians expressed that more training about these new medications would be helpful in their role and that support from the governing bodies would assist in confidence with prescribing the medications:

*‘And I think education, yes, nurses are doing the [clinical diabetes education] course, but it will be good to [have] someone sitting with them [nurses] like a diabetes nurse specialist for the first few months, because it does take time, they took time for me to understand it as well’.* (Nurse-05)

*‘[It would] be helpful for support from organisations like the Ministry of Health or Te Whatu Ora or the Royal College of GPs, to say that it’s okay to do this [the clinical diabetes education course]. Because often doctors are very risk averse in their personality profiles, and they don’t like to try new things. So often, people are very scared or doctors are very scared to try new things. So, if they get support from bigger organisations or educational institutions, saying that it’s okay to try these kinds of things, or if people do trial these things as pilot projects, they can have a very good outcome on doctors to state that. Oh, yeah. Other people have tried it. Nothing’s happened to them. It’s fine’.* (GP-03)

### Benefits of extending the multi-disciplinary primary care team

An unexpected yet significant trend among participants was that many were proactive in trying to support their patients with T2D by offering options for workarounds and solutions to their patients’ challenges. Here, it is important to note that the workaround suggested and enacted by these participants was unique to their clinics and roles, and the resources and knowledge available to each of them. These workarounds were based on need and were aimed at supporting already established clinical and public health practices and attempted to meet the specific financial, social, or geographical challenges that patients face, in relation to effective T2D management. This included referring patients to additional social services that acted as extensions to the primary healthcare team, which was particularly beneficial for patients who could not access general practice due to mobility issues, lack of transport to clinic, or income limitations. One clinician highlighted that their patient was unable to attend clinic so a workaround was finding a service that could visit the patient to assist with effectively managing their T2D:

*‘[There is] a Kaitiaki [health navigator/support] service, run by one of the local PHOs [primary health organisations], and we can refer people there, and they’ll do home visits to help with chronic disease management’.* (GP-07)

Clinicians expressed that addressing the psychosocial aspects of chronic health conditions management was beneficial for patient health, and having support in-house was noted as helpful for clinicians in their roles:

*‘We recently got a…. health improvement practitioner, counsellor. And she’s made a huge difference from the mental health side is actually having someone I can say to them, would you like an appointment tomorrow with that counsellor, whereas previously I had to refer to the community counselling thing through the DHB [District Health Board] where it will be assigned to some counsellor somewhere’.* (GP-02)

A noteworthy and repeatedly suggested point mentioned by participants was the utilisation of a multi-disciplinary clinical and support team to help alleviate workload pressure. The concept of “more staff” was not limited to increasing the clinical workforce but also extended to members of a support team who could help with the non-clinical needs of patients by acting as health-system navigators and advocates due to their past knowledge of the health system. Building a larger multi-disciplinary team of clinical and support staff to overcome some barriers was suggested to be useful not only for clinician workload but for patient care as well:

*‘Functions which can be delegated to less senior staff [that] have not been done [due to time constraints]. So, getting medical students involved, getting kaiawhina [culturally appropriate support staff] on board, getting HIP [Health Improvement Practitioner] staff involved. All those things would be [useful], getting nurses of course, and nurse practitioners and prescribers involved, all those things would be helpful to improve access’.* (GP-03)

The complexities of effective T2D healthcare were highlighted by many participants. Delegating some tasks to non-clinical staff or to junior clinicians was also suggested as a useful strategy, as sometimes patients did not need the doctor, and instead needed non-clinical assistance such as education or advice on what is available to them via social services (for example financial aid) to reduce access barriers. As highlighted by two clinicians:

*‘So, they have a term in the Māori language; it’s called to ‘awhi’ someone; that is to take someone from one place and handhold them [hold their hand] till they reach their destination. So, what they [patients] need, is a, someone who can awhi them from their situation, which is not taking the medication, to guide them, and then get them across to taking and getting their prescription’.* (GP-03)

*‘So, I think it’d be nice if there… Or maybe it’s available already that’s efficient, is looking to, for a health advisor or something, if there’s a place they [patients] can go to get like the right advice or get pointed to the right place or something like that. Maybe that would avoid some barriers…’.* (GP-05)

Having a wider team approach also enabled patients to have longer consultation times with health professionals who had capacity in their roles. This was suggested as useful for improving diabetes outcomes:

*‘And on top of it, we also have a clinical pharmacist, prescriber, on board with us every couple of days, and she has her own sort of appointments. And so, she goes through the diabetes management with the patients. So, she has about 30 to 45 minutes with the patients themselves to really educate them, which is, you know, unheard of 30 to 45 [minutes]’.* (Nurse-02)

Involving team members who can do home visits or be mobile was suggested as useful to reduce access barriers among the ‘hard-to-reach’ patients who need care and provide holistic care (sometimes not just needing diabetes care). As indicated by one GP:

*‘More, more funding available. So, there are more, for example, diabetes, CNSs [chronic care nurse specialists] around, and nurses who can do an outreach home visit or even doctors who can do that. For patients who may not be able to leave the house, funding to trigger services like that would be nice’.* (GP-05)

## Discussion

Three common themes were reported among primary care clinicians experience with optimising T2D management: clinician perspectives on barriers to effective T2D healthcare delivery, clinician views on newly introduced medications, and potential benefits of extending the multi-disciplinary primary care team. Some key barriers identified by clinicians included limited time for appointments, lack of funding, and understaffing, which align with previous studies in NZ (Pullon *et al*., 2009). The need for more clinical staff, especially those specialising in diabetes management, was identified in this study. However, due to T2D being a chronic condition and one that is influenced by lifestyle factors, a need for non-clinical staff was also identified. The healthcare workforce in NZ is currently at a point of crisis (Frizelle, 2022) due to significant staff shortages (Feton *et al*., 2023) and a health-system reform (New Zealand Government, 2022) on the back of a strained COVID-19 period. This has led to greater workload and pressure on clinical staff, with clinicians reportedly feeling burnout (Alkhamees *et al*., 2023). This is concerning as clinician burnout may increase rates of patient medical errors (Hall *et al*., 2016), decrease clinician productivity (Jun *et al*., 2021), increase patient dissatisfaction (Panagioti *et al*., 2018), and ‘moral injury’ as clinical staff are unable to deliver best practice care (Thibodeau *et al*., 2023). Strategies to address clinical workforce shortages are urgently needed in NZ, and in early 2024, government funding was allocated to the development of an additional medical school planned for operation in 2027. However, improvements in the short term are likely to be restricted to improving how healthcare is delivered rather than on the number of staff available. Indeed, clinicians in this study had suggested that T2D patient care may be improved by increasing the employment of non-clinical support staff who can potentially alleviate some of the clinician T2D workload by providing non-clinical lifestyle-related care. These support staff may include health coaches, healthcare assistants, and kaiāwhina who provide culturally appropriate community and mental health support (e.g. by serving to awhi, or guiding and further supporting Māori patients through T2D care). Further, they bridge the gap between patients and health professionals (Te Karu *et al*., 2020) and require significantly less time in tertiary training than clinical staff, potentially resulting in additional help in the interim while increasing the clinical staff workforce over time. This is particularly useful, as clinicians had also identified that patients who lacked stable housing and adequate income faced notable obstacles in accessing healthcare. This highlights the increasing equity gap in healthcare access, which is crucial, especially among patients with comorbidities that may require more frequent visits (Sederer, 2016; Health Navigator NZ, 2021; World Health Organisation, 2022). The addition of support staff, particularly kaiāwhina, to the healthcare team (including mobile and extended primary care teams that utilise additional resources such as dieticians and occupational therapists) (Crosswell *et al*., 2024) has demonstrated improved patient access to medical staff by providing culturally appropriate support and care, working to improve breakdowns in trust between the healthcare space and patients, and ultimately address the inequity faced by Māori and other minoritised patient communities.

Clinicians noted that patient satisfaction and motivation increased with the use of the ‘new’ diabetes medications (SGLT2i and GLP1RA), in NZ. However, some clinicians mentioned they were apprehensive about prescribing these drugs. GPs and nurses identified that support from trusted sources (e.g., the Ministry of Health) and diabetes specialists would be helpful to increase their confidence in prescribing these drugs, and indeed pharmacist prescribers have previously demonstrated that diabetes medication prescribing rates increased in NZ when education and prescribing support is provided to other clinicians (Norman *et al*., 2023). Previous literature has also indicated that clinical inertia could perhaps impact diabetes prescribing, particularly given the constantly changing landscape of pharmaceutical drugs in healthcare (Khunti *et al*., 2013). Tentative adoption of new medications has been reported previously to be due to a lack of clinical confidence and/or awareness (Mason, 2008) though local research has demonstrated that specialised diabetes education is effective when delivered directly to primary care clinicians (Paul *et al.*, 2023). Fostering and nurturing the clinician-patient relationship is crucial for enhancing patient health, and introducing new medications has the potential to strain this bond, thereby posing long-term challenges for clinicians (Damschroder *et al*., 2009). However, NZ-specific clinically-oriented diabetes education has only become widely available online since 2023 (NZSSD, 2021), which does explain, in part, why there has been a gap between the availability of new therapeutic agents and clinical confidence to use them. Bridging this gap is essential, as having a supported, knowledgeable, and confident healthcare workforce can lead to less clinical burnout, improved diabetes health outcomes, reduced inequity, and improved patient quality of life (Shiri & Nikunlaakso, 2023).

In the context of the NZ health system, which is reflective of many healthcare systems worldwide, particularly those in settler colonial nations, it primarily adheres to a Western or Eurocentric framework. Clinicians have observed significant differences between the Western healthcare system’s approach to managing T2D and the perspectives of Indigenous Māori and Pacific populations (Mullane *et al*., 2022). This reported lack of patient satisfaction occurs within a healthcare system that fails to consider the diversity of its patient population (Romana *et al*., 2022). Clinicians have discussed instances where patients, particularly those from non-Western backgrounds (including both Indigenous; Māori and immigrant), may harbour a sense of distrust towards the Western-dominated healthcare system and Western medications with which they may be unfamiliar (Tane *et al*., 2021). This distrust poses an additional barrier within the context of T2D care and necessitates clinicians investing more time in building trust with patients to foster effective care. Incorporating non-Western health worldviews is essential to address inequity, improve patient access to healthcare, develop trust with healthcare providers, and therein improve patient health outcomes (Rolleston *et al*., 2020). This research offers valuable insights into the experiences of primary care clinicians involved in delivering diabetes care, which is crucial for the effectiveness of future diabetes interventions or management strategies. While the study achieved ‘data saturation’ during interviews, it is acknowledged that adopting an Indigenous health lens would likely uncover a broader range of themes among Māori in NZ and this should be explored alongside programmes to better support clinicians to provide optimal healthcare.

### Study limitations and recommendations for future work

While our study has focused on exploring the clinical views and behaviours associated with the management of established T2D, it is important to note that there is also a strong need for patient perspectives and primary prevention of diabetes particularly given the role of diet and exercise changes in the development of the disease (Haw *et al*., 2017). Several studies have reported on the efficacy of primary care-based T2D-prevention programmes (Aziz *et al*., 2015; Sanchez *et al*., 2018), and in NZ, this includes culturally informed programmes to address obesity, diabetes, and cardiovascular disease risk (Barthow *et al*., 2023; Mack *et al*., 2023).

More work is clearly required to address the rising T2D epidemic and its associated health outcomes, and this likely needs to include a multi-pronged approach of preventative care, optimised management, and patient/clinician education. In particular, further studies are required to better understand the current barriers to achieving ongoing and optimised Māori patient-practitioner relationships. In addition, appropriate interventions should be explored/trialled to support improved healthcare access for Māori people, including health economic evaluations as required. Future qualitative evaluations could also look to utilise different recruitment strategies, thereby minimising any bias that may have resulted in our study due to the snowball recruitment and paid incentives used (though koha is an ethical, culturally important (and usual) practice in NZ to thank participants for their time and contribution).

In conclusion, diabetes management appears to remain challenging in NZ, though most clinicians support the use of the newly funded agents empagliflozin and dulaglutide, as they appear to improve both patient satisfaction and clinical outcomes. There continues to be a need for a supportive, multi-disciplinary team to optimise diabetes management, including culturally relevant healthcare workers given the disproportionate burden of T2D in Māori and minority groups. Future research is required to explore improvements in health service delivery and to evaluate the patient voice and costs associated with culturally appropriate delivery of diabetes care.
